# ECD promotes gastric cancer metastasis by blocking E3 ligase ZFP91-mediated hnRNP F ubiquitination and degradation

**DOI:** 10.1038/s41419-018-0525-x

**Published:** 2018-04-30

**Authors:** Song-Hui Xu, Song Zhu, Yanjie Wang, Jin-Zhou Huang, Min Chen, Qing-Xia Wu, Yu-Tian He, De Chen, Guang-Rong Yan

**Affiliations:** 1 Biomedicine Research Center, The Third Affiliated Hospital of Guangzhou Medical University, Guangzhou, 510150 China; 2 Key Laboratory of Protein Modification and Degradation, Guangzhou Medical University, Guangzhou, 510150 China

## Abstract

The human ortholog of the *Drosophila* ecdysoneless gene (ECD) is required for embryonic development and cell-cycle progression; however, its role in cancer progression and metastasis remains unclear. Here, we found that ECD is frequently overexpressed in gastric cancer (GC), especially in metastatic GC, and is correlated with poor clinical outcomes in GC patients. Silencing ECD inhibited GC migration and invasion in vitro and metastasis in vivo, while ECD overexpression promoted GC migration and invasion. ECD promoted GC invasion and metastasis by protecting hnRNP F from ubiquitination and degradation. We identified ZFP91 as the E3 ubiquitin ligase that is responsible for hnRNP F ubiquitination at Lys 185 and proteasomal degradation. ECD competitively bound to hnRNP F via the N-terminal STG1 domain (13-383aa), preventing hnRNP F from interacting with ZFP91, thus preventing ZFP91-mediated hnRNP F ubiquitination and proteasomal degradation. Collectively, our findings indicate that ECD promotes cancer invasion and metastasis by preventing E3 ligase ZFP91-mediated hnRNP F ubiquitination and degradation, suggesting that ECD may be a marker for poor prognosis and a potential therapeutic target for GC patients.

## Introduction

Gastric cancer (GC) is a prevalent malignancy in East Asian countries, including China, and is the second leading cause of cancer-related mortality worldwide, with an overall 5-year survival rate of less than 25%^1,2^. Most GCs are diagnosed clinically at an advanced disease stage and thus present with distant metastases, which are the most important cause of cancer-associated death in GC patients. Although surgical resection is considered the gold standard for treating GC patients, GC patient prognosis remains poor due to the high incidence of tumor recurrence and distant metastasis. Conventional chemotherapy has limited effects on GC, especially metastatic GC. Targeted small molecule or antibody therapies designed to inhibit a specific oncogene are promising therapeutic strategies. Anti-HER2-targeted antibody therapies improve the overall survival of HER2-positive GC patients when combined with chemotherapy; however, HER2-positive patients comprise only 7–17% of GC patients. Therefore, new therapeutic targets are urgently needed.

The ecdysoneless (ECD) gene was originally named by authors studying *Drosophila melanogaster* ECD mutants who exhibited defective development due to reduced production of the steroid hormone, ecdysone, required for insect molting^3^. Subsequent studies showed that the ECD protein is required for cell-autonomous processes in *Drosophila* development and oogenesis^4^. The human ECD homolog was initially identified in a complementation assay conducted to rescue yeast mutants lacking the glycolysis regulation 2 (Gcr2) gene^5^. ECD gene deletion in mouse embryonic fibroblasts led to cell-cycle arrest at the G1/S checkpoint, suggesting ECD is a novel cell-cycle regulator^4,6^. ECD is overexpressed in pancreatic and HER2/ErbB2-overexpressing breast cancers^7,8^. Our previous studies showed that ACK1 promotes GC metastasis through the AKT-POU2F1-ECD pathway and that ECD is a potential key downstream effector of ACK1^1,9^. However, the roles and molecular mechanisms of ECD in cancer progression and metastasis remain unknown.

hnRNP F belongs to the hnRNP family, a large family of RNA-binding proteins that regulate multiple aspects of nucleic acid metabolism, including alternative splicing, transcription, translation, and mRNA stabilization^10^. hnRNP expression is altered in many cancers^10,11^, and these proteins are crucial in cancer cell proliferation, invasion, and metastasis^10,12–15^. hnRNP F/H regulate alternative splicing of the apoptotic regulator, Bcl-x, and the tumor-associated NADH oxidase, ENOX2^16–18^. hnRNP F is a potential marker for colorectal cancer progression^19^; however, the regulatory mechanism of hnRNP F expression upregulation in cancers remains unknown.

Ubiquitination is a well-studied post-translational modification involved in proteasomal degradation, protein–protein interaction, protein trafficking, and protein activity. Protein ubiquitination is mediated by three enzyme families (E1, E2, and E3). Ubiquitination system activity depends on E3 ubiquitin ligase specificity^20–22^. To date, a direct connection between hnRNP F and the ubiquitination pathways remains unobserved, as an hnRNP F-specific E3 ligase that can bind to hnRNP F and induce ubiquitination and proteasomal degradation of hnRNP F has not been identified.

In this study, we found that ECD was overexpressed in GC, especially in metastatic GC, and ECD promotes GC invasion and metastasis by stabilizing hnRNP F. We further found that ZFP91 is the E3 ligase responsible for hnRNP F ubiquitination at Lys 185 and degradation. ECD blocks the interaction between ZFP91 and hnRNP F and the subsequent ubiquitination- and degradation-inducing effects of ZFP91 on hnRNP F by competitively binding to hnRNP F. Our findings indicate that ECD facilitates cancer migration and invasion by stabilizing hnRNP F, and ECD may be used as a novel prognostic GC biomarker, as well as an anti-cancer therapeutic target.

## Results

### ECD overexpression is correlated with aggressive GC phenotypes

**Fig. 1:**
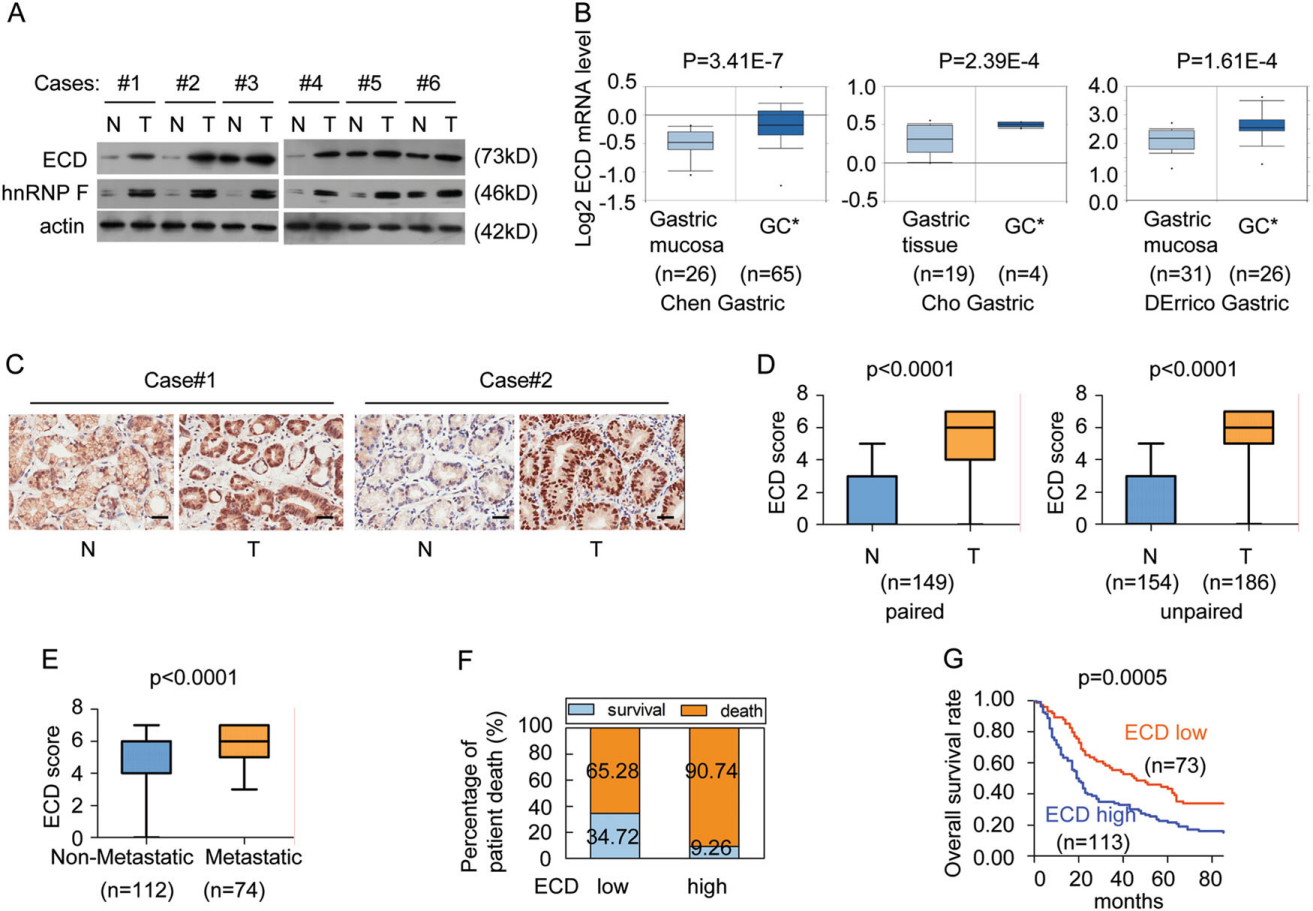
ECD expression is increased in GC and is correlated with a poor prognosis in GC patients. **a** The indicated protein levels in six pairs of primary GC samples (T) and matched adjacent non-tumoral gastric tissue samples (N) were detected. **b** ECD mRNA expression levels were increased in GC compared with those in gastric mucosa, as determined by analyses of three gastric databases from Oncomine. **c** Representative IHC images of ECD expression in the GC tissue specimens and the corresponding N tissue specimens. **d** The differences in ECD expression scores between the GC tissue specimens and the corresponding N tissue specimens are presented as a box plot. **e** ECD expression score differences between the non-metastatic and metastatic GC tissue specimens are presented as a box plot. **f** The associations between ECD levels and patient death percentages were analyzed. **g** Kaplan–Meier survival analysis of patients with GC by their ECD levels

To investigate the role of ECD in gastric progression, we analyzed ECD protein levels in six pairs of primary GC tissue samples and matched adjacent non-tumoral gastric tissue (N) samples. We found elevated ECD protein levels in all GC tissue samples compared to those in the N samples (Fig. 1a). We also investigated ECD mRNA expression in gastric mucosal tissues and GC tissues using three microarray gene expression datasets deposited in the Oncomine database. ECD mRNA levels were higher in gastric intestinal-type adenocarcinoma and gastric adenocarcinoma tissues than in gastric mucosal tissues (Fig. 1b), indicating that ECD expression is upregulated in GC.

To investigate the correlation between ECD levels and prognosis, we performed an extensive tissue microarray analysis of 186 GC tissue samples and 154 adjacent non-tumoral gastric tissue (N) samples (including 149 GC tissue pairs and matched non-tumoral gastric tissues) using an immunohistochemical (IHC) assay (Fig. 1c). We detected higher ECD levels in GC tissues than in N tissues (Fig. 1d), as well as higher ECD levels in metastatic GC tissues than in non-metastatic GC tissues (Fig. 1e).

ECD overexpression was positively associated with pT status, pN status, lymph node metastasis, more advanced histological grades and clinical stage in GC (Table 1). GC patients with high ECD levels were at higher risk for cancer-related death than those with low ECD levels (Fig. 1f, g). The mean overall survival time for GC patients with high ECD levels was 19.5 months, while for GC patients with low ECD levels, it was 45.5 months (*p* = 0.0005, log-rank test). Therefore, ECD overexpression was correlated with poor prognosis in GC patients.

**Table 1:** Correlations between the expression of ECD and clinicopathological features in 186 GC cases

		ECD protein			
	All cases	Low^b^	High^c^	X^2a^	*P* value
Sex				0.118	0.745
Female	66	27	39		
Male	120	46	74		
Age (years)^d^				0.480	0.488
≤60	93	39	54		
>60	92	34	58		
Histological Grade				5.477	0.019
G1–G2	42	23	19		
G3	144	50	94		
Metastasis				6.089	0.014
No	112	52	60		
Yes	74	21	53		
pT status				50.642	0.000
I–II	30	29	1		
III–IV	156	43	113		
pN status^d^				11.611	0.001
I–II	114	56	58		
III	71	17	54		
Clinical stage^d^				30.814	0.000
I–II	65	43	22		
III	119	29	90		

^a^X^2^ test.

^b^ECD expression score is 0–5.

^c^ECD expression score is 6–7.

^d^All case number is less than 186 because the clinicopathological information of some cases is absent or clear.

### ECD promotes GC invasion and metastasis

**Fig. 2:**
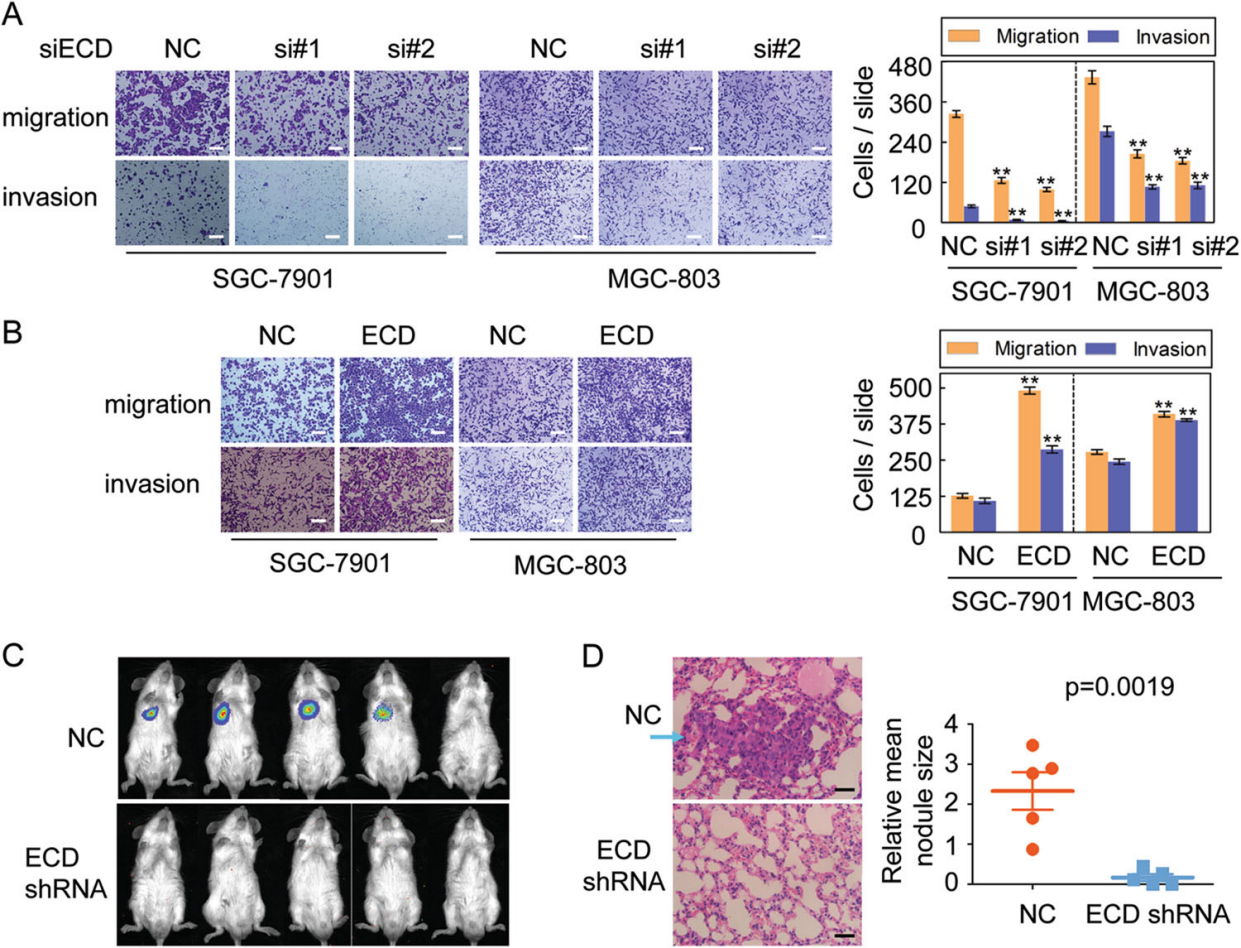
ECD promotes GC migration and invasion in vitro and metastasis in vivo. **a** SGC-7901 and MGC-803 cells were transfected with two anti-ECD siRNAs, and cell migration and invasion abilities were determined. **b** SGC-7901 and MGC-803 cells were transfected with ECD plasmids, and cell migration and invasion abilities were determined. **c** NOD-SCID mice were transplanted with the indicated luciferase-labeled SGC-7901 cells (2 × 10^6^ cells/mouse) via tail vein injections. Mice were assessed at 2 months post-transplantation (*n* = 5). **D** Tumor nodule size was decreased in mice pulmonary metastases composed of SGC-7901 cells with ECD knockdown. Histological analysis of pulmonary metastases in the mouse model was determined by HE staining (left panel). The relative mean tumor nodule size was quantified (right panel) (*n* = 5). Results in (**a**) and (**b**) are shown as means ± SEM of three independent experiments. *p < 0.05 or **p < 0.01 was considered statistically significant

To investigate the role of ECD in GC invasion and metastasis, we either silenced or overexpressed ECD in two GC cell line models. ECD silencing suppressed GC cell migration and invasion in SGC-7901 and MGC-803 cells (Fig. 2a), while ECD overexpression promoted GC cell migration and invasion in those cells (Fig. 2b).

Smaller metastatic nodules developed in mouse lungs after injection with luciferase-tagged SGC-7901 cells with stable silencing of ECD expression than those in mouse lungs after injection with SGC-7901 cells (Fig. 2c). Smaller metastatic lung nodules were also confirmed by histological analysis (Fig. 2d). Collectively, these results indicate that ECD promotes GC cell migration and invasion in vitro and metastasis in vivo.

### ECD interacts with hnRNP F via the SGT1 domain

**Fig. 3:**
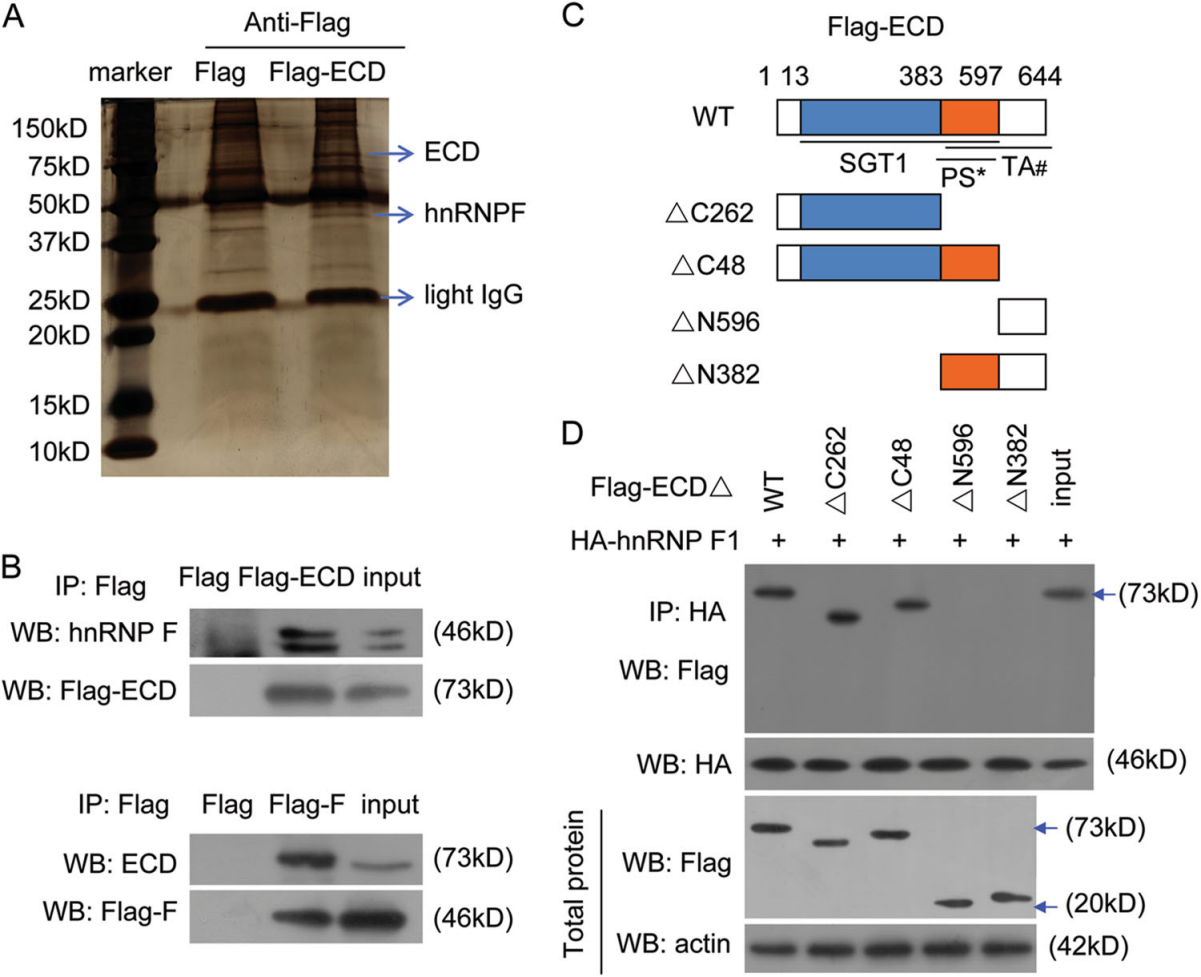
ECD interacts with hnRNP F through the N-terminal SGT1 domain. **a** Proteins that interacted with Flag-ECD were identified by Co-IP and mass spectrometry assays. **b** SGC-7901 cells were transfected with Flag-ECD (upper panel) or Flag-hnRNP F (Flag-F) (low panel) plasmids, and the resulting Flag-ECD or Flag-F complexes were co-immunoprecipitated by anti-Flag antibodies. hnRNP F and ECD presence in these complexes was detected. **c** ECD wild-type and mutation construct diagrams and the different domains. PS^*^ indicates predicted structure, and TA^#^ indicates transactivation domain. **d** The indicated Flag-ECD mutation constructs and HA-hnRNP F were co-transfected into HeLa cells, and the resulting HA-hnRNP F complexes were co-immunoprecipitated by anti-HA antibodies. Flag-ECD mutants were detected using anti-Flag antibodies. Results in (**a**) and (**b**) are shown as means ± SEM of three independent experiments

To investigate the mechanism by which ECD promotes cancer invasion and metastasis, we identified the proteins that interact with ECD by performing Co-IP and mass spectrometry analyses (Fig. 3a). We identified that hnRNP F might interact with ECD. We further confirmed that ECD interacts with hnRNP F (Fig. 3b).

ECD consists of a conserved SGT1 domain and transactivation region^5^. To determine which domains interact with hnRNP F, we generated truncated ECD constructs with an N-terminal Flag tag (Fig. 3c). When these constructs were co-expressed with HA-hnRNP F in cells, only the constructs containing the ECD N-terminal STG1 domain (13-383aa), but no other ECD regions, could interact with hnRNP F, indicating that the N-terminal STG1 domain is essential for hnRNP F binding (Fig. 3d).

### ECD increases hnRNP F protein levels by inhibiting hnRNP F polyubiquitination and degradation

**Fig. 4:**
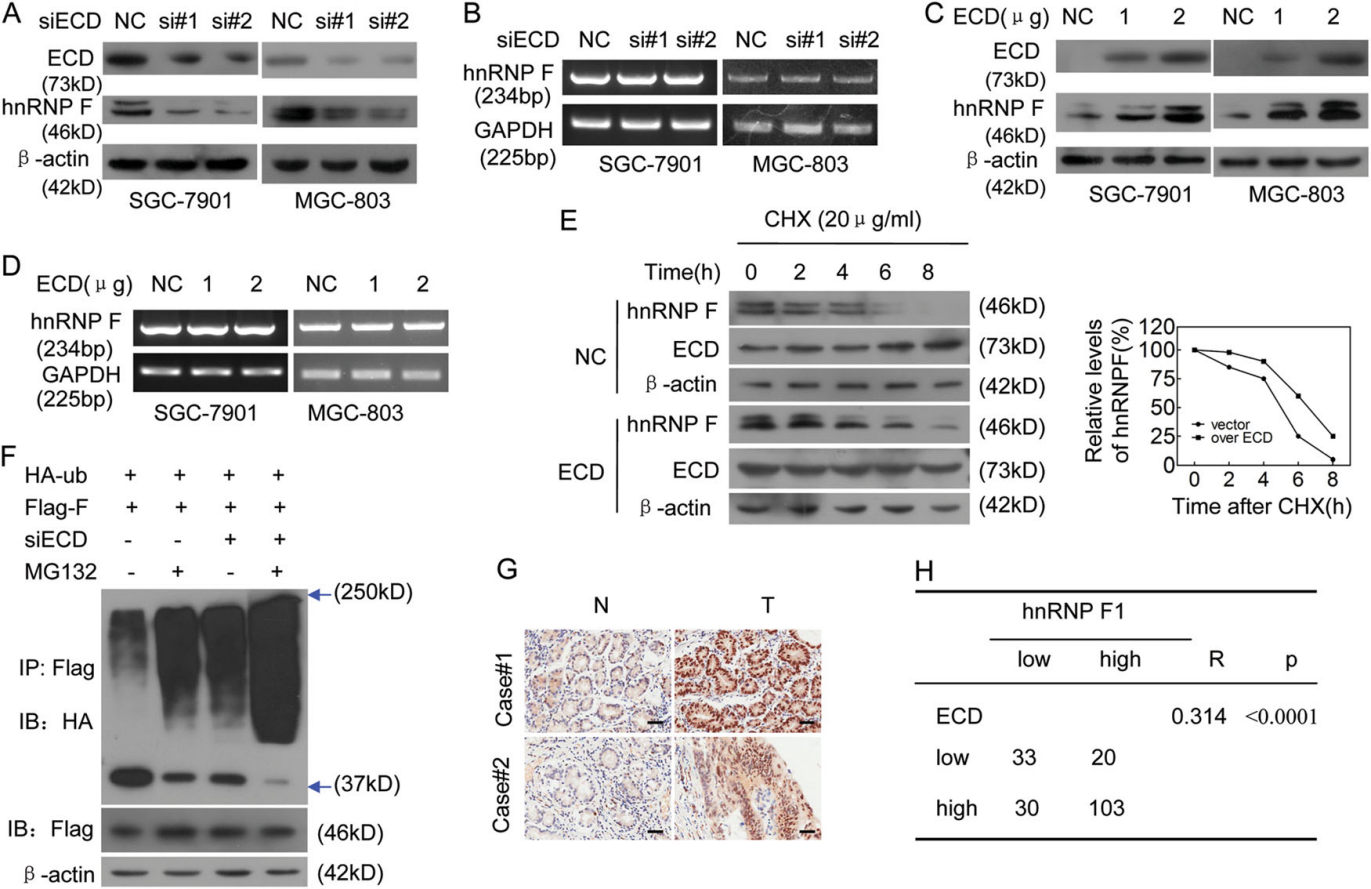
ECD upregulates hnRNP F protein expression levels by stabilizing hnRNP F. **a**, **b** SGC-7901 and MGC-803 cells were transfected with two anti-ECD siRNAs, and the protein (**a**) and mRNA (**b**) levels of the indicated genes were determined. **c**, **d** SGC-7901 and MGC-803 cells were transfected with ECD plasmids at the indicated doses, and the protein (**c**) and mRNA (**d**) levels of the indicated genes were determined. **e** SGC-7901 cells were transfected with Flag-ECD plasmids for 36 h, then incubated with cycloheximide (CHX) for the indicated times. The indicated proteins were analyzed (left panel), and the hnRNP F protein band densities at each time point were normalized to those of β-actin and converted into percentages using 100% as the value of the zero time point (right panel). **f** SGC-7901 cells were co-transfected with the indicated plasmids and siRNAs for 36 h before being treated with or without MG132 (10 μM) for 4 h. Flag-hnRNP F protein was immunoprecipitated by anti-Flag antibody. The ubiquitin in Flag-hnRNP F protein was detected using anti-HA antibodies. **g** Representative IHC images of hnRNP F expression in the GC samples and corresponding N samples. **h** hnRNP F protein levels were positively correlated with ECD protein levels in the GC samples

How does ECD affect hnRNP F given that ECD can bind to hnRNP F? To address this, we investigated the effects of ECD on hnRNP F protein and mRNA levels. ECD silencing decreased hnRNP F protein levels, but not hnRNP F mRNA levels (Fig. 4a, b), while ECD overexpression increased hnRNP F protein levels, but not hnRNP F mRNA levels, in a dose-dependent manner (Fig. 4c, d), indicating that ECD upregulated hnRNP F protein levels at the post-transcriptional level.

Because ECD may upregulate hnRNP F protein levels, and the ubiquitination/proteasome pathway is the quickest known mechanism through which proteins are irreversibly degraded^20,23,24^, we surmised that ECD upregulated hnRNP F by preventing its ubiquitination. To test this hypothesis, we determined the effect of ECD overexpression on the half-life of hnRNP F. As shown in Fig. 4e, ECD overexpression resulted in a longer hnRNP F half-life. We also performed an in vivo ubiquitination assay, which showed that silencing ECD in the presence of MG132 (a specific proteasome inhibitor) increased hnRNP F polyubiquitination levels (Fig. 4f). Collectively, our results strongly indicate that ECD inhibits hnRNP F protein polyubiquitination and proteasomal degradation, thus increasing hnRNP F protein levels.

Furthermore, we investigated the correlation between ECD and hnRNP F protein levels in GC tissues. We found that hnRNP F protein levels were upregulated in GC tissues compared with those in matched N tissues, consistent with the results of the ECD expression experiments (Fig. 1a). Extensive tissue microarray analysis showed that hnRNP F protein levels were higher in GC tissues than in N tissues (Fig. 4g); however, hnRNP F mRNA levels were unchanged between GC tissues and N gastric tissues (Supplementary Fig. S1). ECD protein levels were also positively correlated with hnRNP F protein levels in GC tissue samples (*R* = 0.314, *p* < 0.0001) (Fig. 4h). These findings indicate that ECD increases hnRNP F protein levels by stabilizing hnRNP F.

### ECD stimulates GC migration and invasion by stabilizing hnRNP F

Next, we investigated the role of hnRNP F in ECD functions. We found that silencing of hnRNP F inhibited GC cell migration and invasion, similar to the effects induced by ECD knockdown (lane 3 in Supplementary Fig. S2B). And silencing hnRNP F attenuated the enhanced migration and invasion induced by ECD overexpression (Supplementary Fig. S2), indicating that ECD promotes GC migration and invasion by regulating hnRNP F stability.

### ZFP91 is an E3 ubiquitin ligase responsible for ubiquitinating hnRNP F

**Fig. 5:**
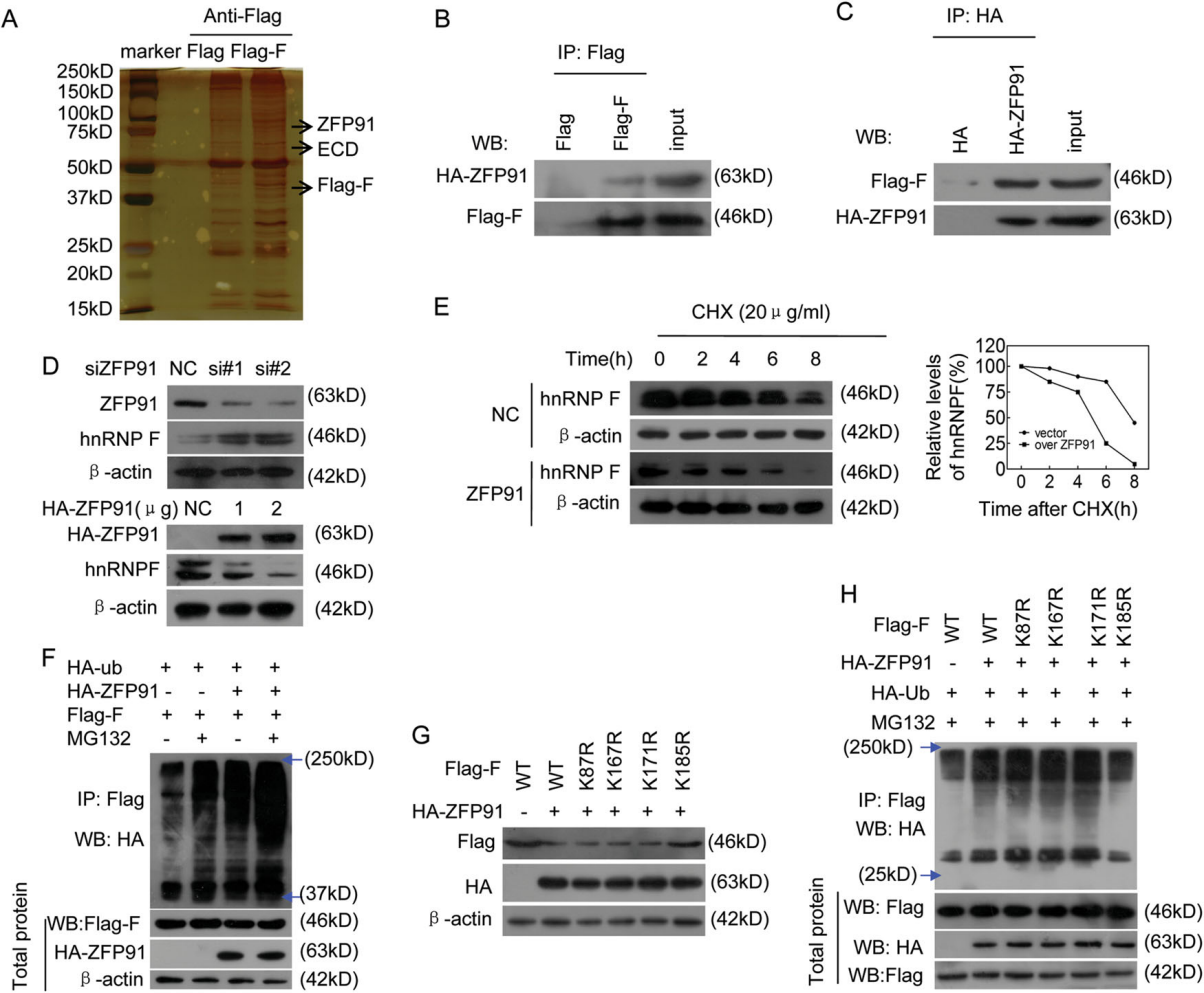
ZFP91 E3 ligase interacts with hnRNP F and regulates hnRNP F protein ubiquitination at Lys 185 and degradation. **a** The proteins that interacted with Flag-hnRNP F (Flag-F) were identified by combining Co-IP and mass spectrometry assays. E3 ligase ZFP91 interacted with hnRNP F. **b**, **c** The Flag-F plasmid and HA-ZFP91 vector were co-transfected into SGC-7901 cells, and the resulting Flag-hnRNP F (**b**) or HA-ZFP91 (**c**) complexes were co-immunoprecipitated by anti-Flag antibodies. ZFP91 or hnRNP F presence in these complexes was detected using HA or Flag antibodies, respectively. **d** SGC-7901 cells were transfected with two anti-ZFP91 siRNAs (upper panel) or ZFP91 plasmids (low panel), and the indicated protein levels were determined. **e** SGC-7901 cells were transfected with HA-ZFP91 plasmids for 36 h and then incubated with CHX for the indicated times. The half-life of the hnRNP F protein was analyzed as described in Fig. 4e. **f** Cells were co-transfected with the indicated plasmids for 36 h, then treated with or without MG132 (10 μM) for 4 h. The polyubiquitination pattern of hnRNP F was analyzed as described in Fig. 4f. **g** The indicated Flag-hnRNP F ubiquitin-lysine mutants and HA-ZFP91 plasmid were co-transfected into HeLa cells, and the indicated protein expression levels were detected. **h** The indicated Flag-hnRNP F mutants and HA-ZFP91 plasmid were co-transfected into GC cells, and cell migration and invasion ability were assessed

As ECD is not an E3 ligase, and the E3 ligase responsible for hnRNP F ubiquitination remains unknown, we sought to identify the E3 ligase that regulates hnRNP F protein ubiquitination and degradation. E3 ligases must bind to their substrates to facilitate their ubiquitination. Thus, we identified the proteins that interacted with hnRNP F by performing Co-IP and a mass spectrometry assay to identify the E3 ligase responsible for ubiquitinating hnRNP F. We found that ZFP91 is the potential E3 ligase because it can bind to hnRNP F (Fig. 5a). We further confirmed that ZFP91 interacts with hnRNP F (Fig. 5b, c), thus suggesting that ZFP91 may be the E3 ligase responsible for polyubiquitinating hnRNP F.

To determine whether ZFP91 is the E3 ligase responsible for hnRNP F ubiquitination, we investigated the effects of ZFP91 on hnRNP F mRNA and protein levels, half-life and ubiquitination. We found that silencing ZFP91 increased hnRNP F protein levels, but not hnRNP F mRNA levels, while ectopically expressing ZFP91 decreased hnRNP F protein levels, but not hnRNP F mRNA levels (Fig. 5d, Supplementary Fig. S3). Moreover, ZFP91 overexpression resulted in a shorter hnRNP F protein half-life (Fig. 5e), and our in vivo ubiquitination assay results showed that ZFP91 overexpression increased hnRNP F polyubiquitination levels in the presence of the specific proteasome inhibitor, MG132 (Fig. 5f). This indicates that ZFP91 is the E3 ligase responsible for ubiquitinating and degrading hnRNP F.

### ZFP91 induces hnRNP F ubiquitination at Lys 185

To identify the specific ubiquitination modification lysine sites in the hnRNP F protein, we searched the PhosphoSitePlus post-translational modification resource. Four potential ubiquitination sites at lysine residues were found in the hnRNP F protein (Supplementary Fig. S4A). We subsequently generated hnRNP F mutants in which these lysine residues were replaced with arginine. We found that ZFP91 overexpression did not change the protein levels in the hnRNP F mutant with K185R, but it decreased the protein levels in the hnRNP F mutants with K87R, K167R and K171R (Fig. 5g). ZFP91 overexpression also increased the hnRNP F mutant polyubiquitination levels with K87R, K167R, or K171R, but not in the mutant with K185R (Fig. 5h). These data indicate that hnRNP F ubiquitination at Lys 185 was regulated by ZFP91 E3 ligase.

We further investigated the effects of hnRNP F ubiquitination at Lys 185 on ZFP91-mediated cancer cell migration and invasion. We found that ZFP91 overexpression inhibited GC cell migration and invasion, thus exerting effects that contrast with those of hnRNP F (Supplementary Fig. S4B). ZFP91 co-expression also attenuated the enhanced migration and invasion induced by overexpressing the hnRNP F mutants with K87R, K167R, or K171R, as ZFP91 polyubiquitinated these hnRNP F mutants and facilitated their subsequent degradation by the proteasome; however, ZFP91 co-expression did not block the enhanced migration and invasion induced by overexpressing the hnRNP F mutant with K185R because this protein was not polyubiquitinated and was degraded by ZFP91 (Supplementary Fig. S4B). Collectively, our results indicate that ZFP91 polyubiquitinated hnRNP F at Lys 185.

### ECD blocks ZFP91 from binding to hnRNP F

**Fig. 6:**
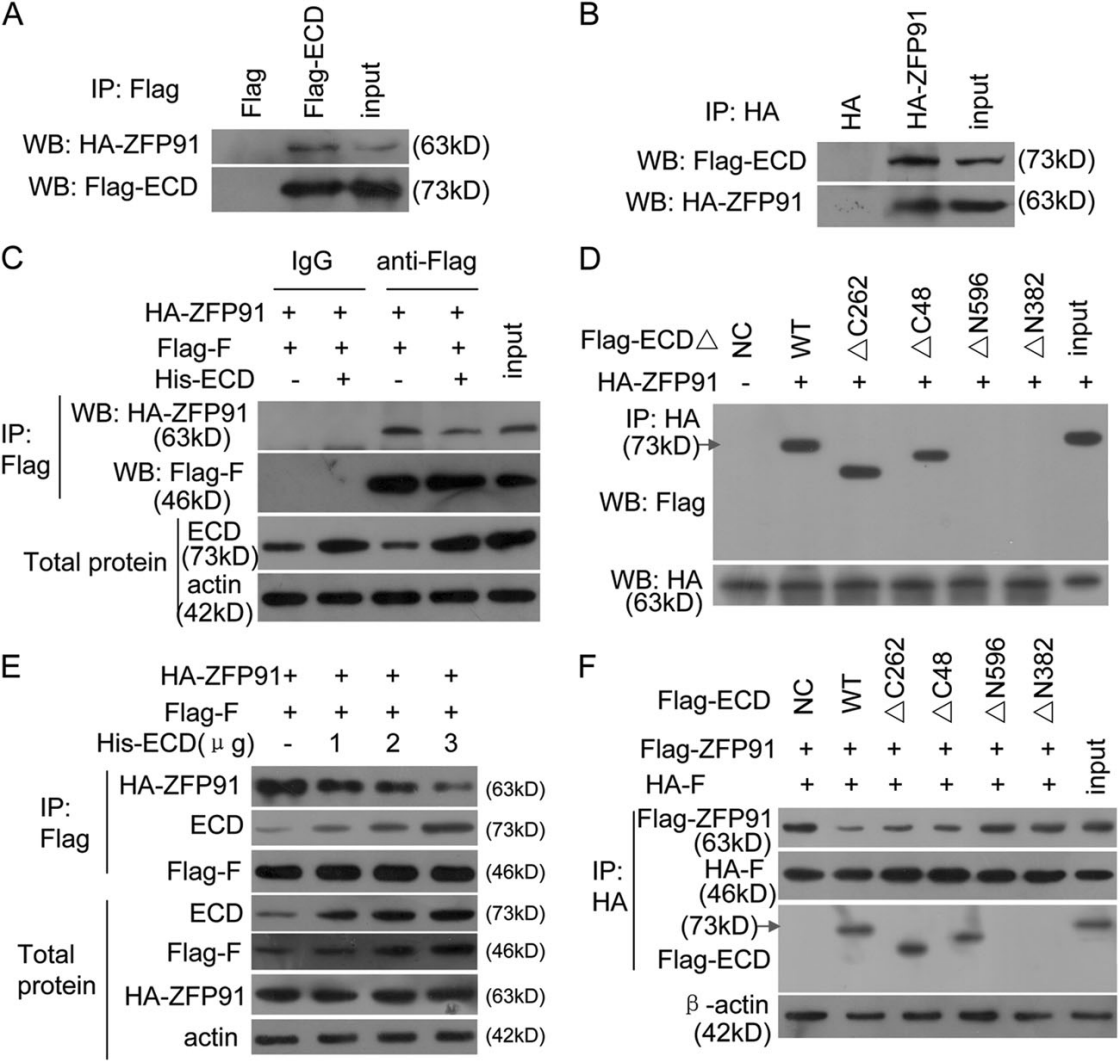
ECD blocks the ZFP91 and hnRNP F interaction. **a**, **b** The Flag-ECD plasmid and HA-ZFP91 vector were co-transfected into SGC-7901 cells, and the resulting Flag-ECD (**a**) or HA-ZFP91 (**b**) complexes were co-immunoprecipitated by anti-Flag antibodies. ZFP91 or hnRNP F presence was detected using anti-HA or Flag antibodies, respectively. **c** The Flag-ECD mutants and HA-ZFP91 plasmids were co-transfected into HeLa cells, and the resulting HA-ZFP91 complexes were co-immunoprecipitated by anti-HA antibodies. Presence of Flag-ECD mutants in these complexes was detected by anti-Flag antibodies. **d** The indicated plasmids were co-transfected into HeLa cells, and the resulting Flag-hnRNP F complexes were co-immunoprecipitated by anti-Flag antibodies. ZFP91 presence in this complex was detected using anti-HA antibodies. **e** The indicated plasmids and ECD plasmids were co-transfected into HeLa cells at the indicated doses, and the resulting Flag-hnRNP F complexes were co-immunoprecipitated by anti-Flag antibody. ZFP91 presence in this complex was detected using anti-HA antibodies. **f** The Flag-ECD mutants and indicated plasmids were transfected into HeLa cells, and the resulting HA-hnRNP F complexes were co-immunoprecipitated by anti-HA antibodies. ZFP91 presence in this complex was detected using anti-Flag antibodies

We further investigated, and subsequently found, an interaction between ECD and ZFP91 (Fig. 6a, b). The constructs containing the ECD N-terminal STG1 domain (13-383aa), but not other ECD regions, retained interactions with ZFP91 (Fig. 6c), suggesting that the same ECD domain that bound to hnRNP F interacted with ZFP91.

Because ECD binds to ZFP91 and hnRNP F, and ZFP91 binds to hnRNP F, we surmised that ECD inhibits the interaction between ZFP91 and hnRNP F. We co-transfected ZFP91, hnRNP F, and ECD plasmids into HeLa cells and assessed the protein interactions. We found that ECD overexpression dose-dependently blocked the ZFP91 and hnRNP F interactions (Fig. 6d, e). Furthermore, we found that the constructs containing the ECD N-terminal STG1 domain (13-383aa), but not other ECD regions, blocked the ZFP91 and hnRNP F interactions (Fig. 6f). This indicates that ECD blocked ZFP91 from binding to hnRNP F through the N-terminal STG1 domain.

### ECD blocks ZFP91-mediated hnRNP F ubiquitination and degradation

**Fig. 7:**
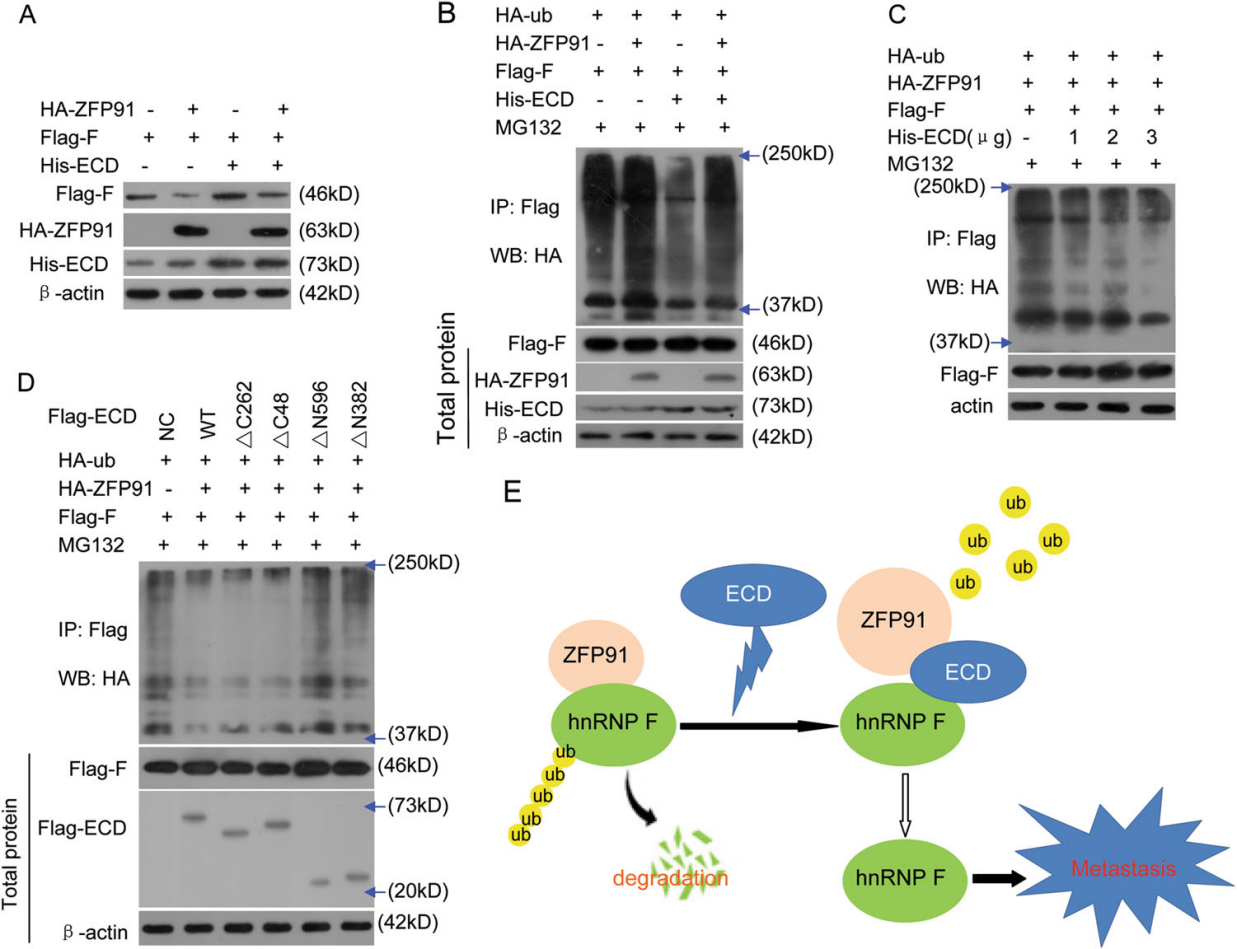
ECD blocks ZFP91 E3 ligase-mediated hnRNP F protein ubiquitination and degradation. **a** The indicated plasmids were transfected into HeLa cells, and the indicated protein expression levels were determined. **b** HeLa cells were co-transfected with the indicated plasmids for 36 h, then treated with 10 μM MG132 for 4 h. The hnRNP F polyubiquitination pattern was analyzed as described in Fig. 4f. **c** His-ECD vectors and the indicated plasmids were transfected into HeLa cells at the indicated doses. The hnRNP F polyubiquitination pattern was analyzed as described in Fig. 4f. **d** The indicated Flag-ECD mutants and plasmids were transfected into HeLa cells. The hnRNP F polyubiquitination pattern was analyzed as described in Fig. 4f. **e** A proposed model illustrating that ECD promotes cancer metastasis by blocking the ZFP91 E3 ligase from binding to hnRNP F, and the subsequent ubiquitination and degradation of hnRNP F by ZFP91 E3 ligase

Because ZFP91 is an E3 ubiquitin ligase that acts on hnRNP F, and ECD blocks the interaction between ZFP91 with hnRNP F and inhibits hnRNP F degradation, we surmised that ECD blocks ZFP91-mediated hnRNP F ubiquitination and degradation. We co-transfected ZFP91, hnRNP F, and ECD plasmids into HeLa cells and investigated hnRNP F protein and ubiquitination levels. ECD reversed the decreased hnRNP F protein levels induced by ZFP91 overexpression (Fig. 7a). In addition, ECD dose-dependently blocked the enhanced hnRNP F ubiquitination levels induced by ZFP91 overexpression (Fig. 7b, c).

We further investigated whether the ECD N-terminal STG1 domain can block ZFP91-mediated hnRNP F ubiquitination, as this domain blocked the interaction between ZFP91 and hnRNP F. The mutants containing the ECD N-terminal STG1 domain exerted effects similar to wild-type ECD and blocked the increased hnRNP F polyubiquitination levels induced by ZFP91, while the mutants containing other ECD regions did not (Fig. 7d). This indicates that ECD blocks ZFP91-mediated hnRNP F ubiquitination and degradation through the N-terminal STG1 domain.

## Discussion

We found that ECD induces cancer invasion and metastasis, and we elucidated the novel mechanism underlying ECD’s effect on cancer progression. Specifically, we found that ECD stabilizes hnRNP F, by blocking the interaction between ZFP91 and hnRNP F and the subsequent ubiquitination and degradation of hnRNP F by ZFP91. ZFP91 is an E3 ubiquitin ligase that ubiquitinates hnRNP F at Lys 185.

Initial studies demonstrated that the Drosophila ECD is required for embryonic development^4,7,8,25^. Here, we demonstrated that human ECD homolog levels were significantly increased in GC tissues, especially in metastatic GC tissues, compared with those in adjacent non-tumoral gastric tissues. ECD mRNA levels were also increased in GC tissues compared with those in the gastric mucosa tissues in three HCC datasets deposited in the Oncomine database. These results indicated that ECD upregulation in GC was induced at the transcriptional level, consistent with our previous study in which we observed that ACK1 stimulated the transcription factor POU2F1 to induce ECD transcription^1^. ECD overexpression was significantly associated with aggressive GC phenotypes, as GC patients with high ECD levels exhibited higher death rates and shorter survival times than GC patients with low ECD levels. Our findings suggest that ECD may be a novel independent prognostic factor in GC patients. Silencing ECD significantly suppressed GC metastasis and invasion in vitro and in vivo, indicating that ECD may be a novel therapeutic target for GC.

Previous studies reported that ECD promotes cell proliferation by regulating RB/E2F pathway-dependent cell-cycle progression and GLUT4-dependent glycolysis in breast and pancreatic cancer, respectively^4,8^. Here, we elucidated a novel mechanism underlying the effects of ECD on GC invasion and metastasis. We showed that ECD promoted GC metastasis and invasion by stabilizing hnRNP F. We found that ECD bound to hnRNP F to prevent E3 ligase ZFP91 from interacting with hnRNP F, thus blocking ZFP91-mediated hnRNP F polyubiquitination at Lys 185 and increasing hnRNP F protein levels to promote cancer metastasis and invasion. Previous studies showed that hnRNP F mRNA levels were increased by OKI-6 in myelinating glia^26^. In this study, we discovered a novel mechanism through which hnRNP F is upregulated by ECD blocking the ubiquitin/proteasome pathway. The double bands for hnRNP F were detected; the possible causes include the different isoform of hnRNP F and the cleaved hnRNP F.

ZFP91 is a novel E3 ligase that activates the NF-κB-inducing kinase (NIK) via Lys63-linked ubiquitination in the noncanonical NF-κB signaling pathway^27^. Here, we noted that in addition to activating substrates via ubiquitination, ZFP91 also interacted with and promoted the ubiquitination of hnRNP F at Lys 185, as well as its subsequent degradation by the proteasome. Here, we report for the first time that ZFP91 is an E3 ligase that ubiquitinates and degrades hnRNP F.

In conclusion, we found that ECD is overexpressed in GC, especially in metastatic GC, and that ECD overexpression is correlated with a malignant phenotype and poor prognosis for GC patients. We elucidated the novel molecular mechanism underlying the effects of ECD in cancer progression, as we determined that ECD promotes invasion and metastasis by stabilizing hnRNP F. We further demonstrated that ECD competitively bound to hnRNP F through the N-terminal STG1 domain to prevent ZFP91-mediated hnRNP F ubiquitination and degradation. We discovered the E3 ligase, ZFP91, which is responsible for hnRNP F ubiquitination and degradation at Lys 185. Our findings indicate that ECD may be a new prognostic factor and potential anti-cancer target for GC.

## Materials and Methods

### Cell culture and tissue samples

HeLa cells were obtained from and authenticated by ATCC. The GC cell lines, SGC-7901 and MGC-803, were obtained from and authenticated by isoenzyme assay by the Institute of Biochemistry and Cell Biology, Chinese Academy of Sciences (Shanghai, China)^28^. The cells were cultured as previously described^1,9^. All cell lines were treated with Plasmocin and tested with Mycoplasma PCR Detection Kit (Sigma-Aldrich, USA). Primary GC tissue samples and matched adjacent non-tumoral gastric tissue samples were collected at The Third Affiliated Hospital of Guangzhou Medicine University. Samples with a clear pathological diagnosis were selected and obtained from GC patients who had not received preoperative anti-cancer treatment. Informed consent was obtained from each patient who participated in the study, and tissue sample collection was approved by the Internal Review and Ethics Boards of The Third Affiliated Hospital of Guangzhou Medicine University. Tissue microarray chips containing GC tissue samples and matched adjacent non-tumoral gastric tissue samples along with their corresponding clinicopathological data were obtained from Shanghai OUTDO Biotech Co., Ltd. (Shanghai, China).

### Immunohistochemistry (IHC) staining assay

IHC staining assays were performed on tissue microarray chips with anti-ECD and anti-hnRNP F antibodies, as previously described, with minor modifications^29^. All IHC staining results were assessed by two independent pathologists blinded to the sample origins and the corresponding patient outcomes. ECD and hnRNP F expression was evaluated using a previously described semi-quantitative German scoring system, which assesses protein expression based on staining intensity and area. Scores of 0–5 indicated low ECD and hnRNP expression (low) in the GC tissue, and scores of 6–7 indicated high ECD and hnRNP expression (high) in the GC tissue.

### Migration and invasion assays

In vitro migration and invasion assays were performed using Transwell chambers, as previously described^1^.

### Western blotting

Western blotting was performed as previously described^1^.

### Experimental in vivo metastasis model

Male NOD-SCID mice (aged 4–5 weeks) were obtained from Charles River Laboratories in China (Beijing). The mice were bred and maintained under defined conditions at the Animal Experiment Center of the College of Medicine (SPF grade), Jinan University. The mice were randomized to allocate into experimental groups by the researchers blinded to the subject outcomes. In vivo metastatic assays were performed as previously described, with minor modifications^30^. Briefly, five NOD-SCID mice in each experimental group were injected with luciferase-labeled SGC-7901-Luc-NC or SGC-7901-Luc-ECD shRNA-transduced cells (2 × 10^6^ cells in 0.2 ml^−1^ of PBS) via their tail veins. The resultant metastatic foci in the lungs were visualized 2 months after tumor implantation. Animal experiments were approved by the Laboratory Animal Ethics Committee of Jinan University and conformed to the legal mandates and national guidelines for the care and maintenance of laboratory animals.

### Co-immunoprecipitation (Co-IP)

Cells were transfected with Flag-ECD, Flag-hnRNP F (Flag-F), HA-ZFP91, or Flag (negative control) vectors for 48 h. Co-IP was subsequently performed using anti-Flagor HA antibodies (MBL), and the immune complexes were captured on protein A/G agarose beads (Santa Cruz, USA) and separated by SDS-PAGE. The SDS-PAGE gels were stained with silver. Protein expression was detected by western blotting using the indicated antibodies.

### Protein identification by mass spectrometry

The differential gel bands and their corresponding negative gel bands were excised and digested using in-gel trypsin. The extracted peptide mixtures were analyzed using nano-LC-MS/MS, as previously described^31^. Proteins were identified using the Mascot (v2.3.02) program against the Uniprot human protein database (released Dec. 2014) using the default settings. Protein scores were ≥40, and unique peptides were ≥2.

### In vivo ubiquitination assay

This procedure was performed as previously described^22^. Briefly, the cells were co-transfected with the indicated plasmids for 36 h, then treated with 10 μM MG132 for 4 h prior to harvesting. The cells were lysed in RIPA buffer with protease inhibitor cocktail (Roche, Switzerland). Flag-hnRNP F was immunoprecipitated using anti-Flag antibodies and protein A/G agarose beads. Polyubiquitinated hnRNP F was detected using anti-HA antibodies.

### Statistical analysis

Two-tailed Student’s *t*-tests or Mann–Whitney *U* tests were used for comparisons between two groups. Survival analysis was performed using the Kaplan–Meier method and the log-rank test. Statistical analyses were performed using Prism 5.0 software. Data are presented as the mean ± SEM unless otherwise stated. *p < 0.05 or **p < 0.01 was considered statistically significant.

## Electronic supplementary material

Supplementary information
