# Supplementary material for: ECD promotes gastric cancer metastasis by blocking E3 ligase ZFP91-mediated hnRNP F ubiquitination and degradation
Source: Cell Death Dis. 2018 Apr 30;9(5):479. doi: 10.1038/s41419-018-0525-x (PMC5924763; doi:10.1038/s41419-018-0525-x)
Supplement: Supplementary file 1 — Supplementary information [file 41419_2018_525_MOESM1_ESM.pdf]

Supplementary information

Supplementary Figures

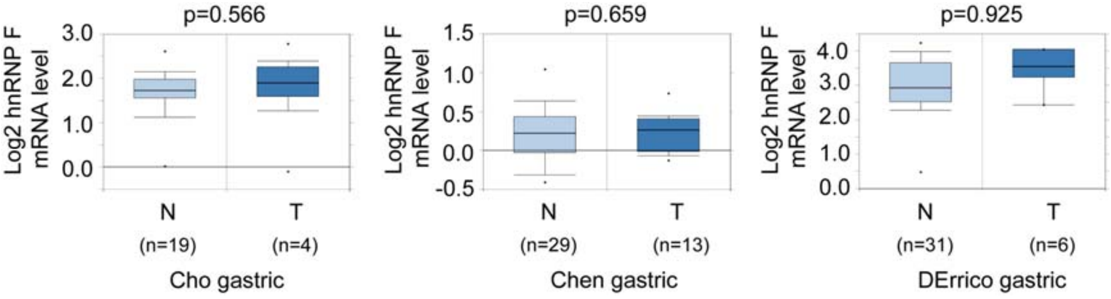

Figure S1. The hnRNP F mRNA levels were not changed between normal gastric tissues (N) and gastric tissues (T). The hnRNP F mRNA expression in gastric mucosal tissues and GC tissues was analyzed using three microarray gene expression datasets deposited in the Oncomine database.

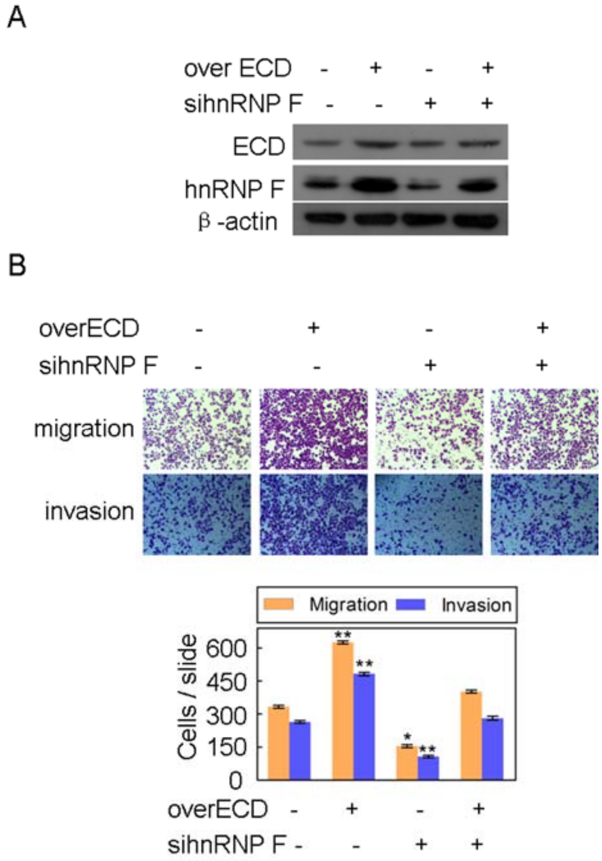

Figure S2. ECD promotes GC migration and invasion by stabilizing hnRNP F. (A, B) The Flag-ECD plasmid and anti-hnRNP F siRNA were co-transfected into SGC-7901 cells, after which the expression levels of the indicated proteins (A) and the migration and invasion ability of the indicated cell line (B) were determined. Results in (B) are shown as means ± SEM of three independent experiments. *p<0.05 or **p<0.01 was considered statistically significant.

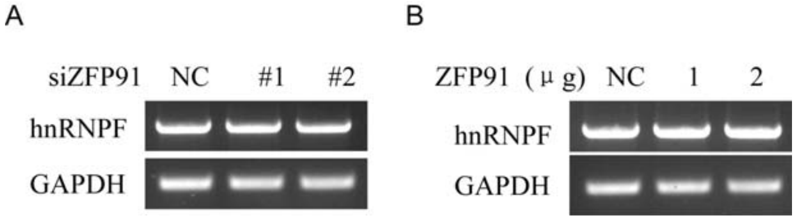

Figure S3. ZFP91 E3 ligase did not change hnRNP F mRNA levels. (A, B) SGC-7901 cells were transfected with two anti-ZFP91 siRNAs (A) or ZFP91 plasmids at the indicated dose (B), after which hnRNP F mRNA levels were determined by RT-PCR.

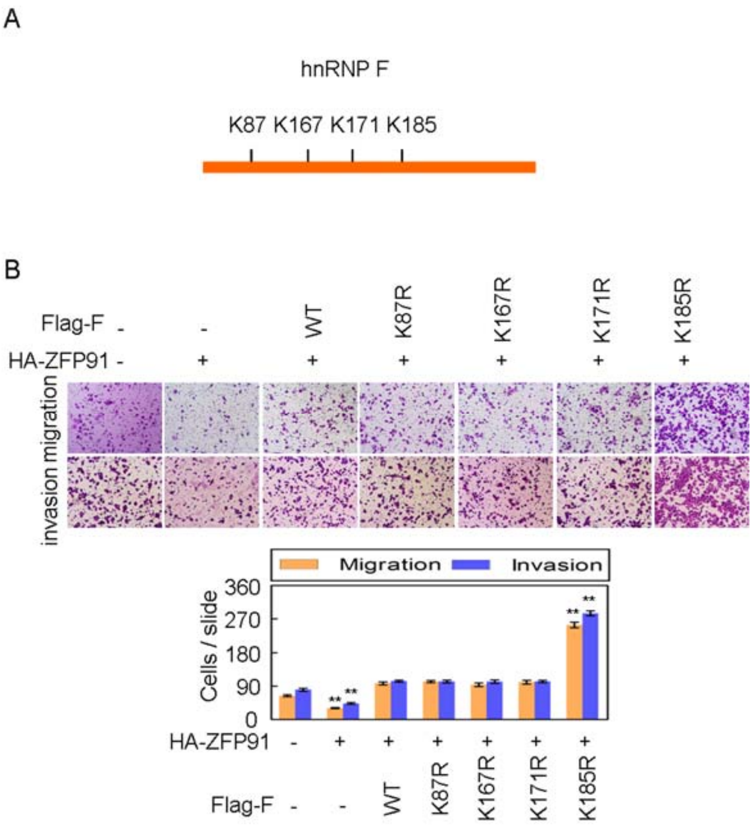

Figure S4. ZFP91 inhibits GC cell migration and invasion by ubiquitinating the hnRNP F protein at Lys 185. (A) A schematic diagram of the ubiquitination sites in the hnRNP F protein. (B) The indicated Flag-hnRNP F mutants and HA-ZFP91 plasmid were co-transfected into GC cells, after which cell migration and invasion ability was assessed. Results in (B) are shown as means ± SEM of three independent experiments. *p<0.05 or **p<0.01 was considered statistically significant.

Supplementary Materials and Methods

RNA interference

SiRNAs against the ECD, hnRNP F and ZFP91 genes and corresponding scrambled siRNAs (GenePharma) were transfected into GC cells with RNAiMAX (Invitrogen) for 48 h (unless otherwise stated). The cells were subsequently harvested for western blotting and migration and invasion assays unless otherwise stated.

Plasmid construction

Expression plasmids containing pCMV-Flag-ECD, pCMV-Flag-hnRNP F (Flag-F), pcDNA3.1-HA-hnRNP F, pCMV-Flag-ZFP91 and pcDNA3.1-HA-ZFP91 were constructed as previously described (1), and Flag-tagged ECD mutants containing different ECD domains were produced as previously described (1). The HA-tagged ubiquitin (HA-ub) plasmid was kindly provided by Prof. Tiebang Kang from the State Key Laboratory of Oncology in South China. Mutations were produced using a QuikChange Site-Directed Mutagenesis Kit (Stratagene) and validated by DNA sequencing.

Construction of cell lines with stable silenced ECD

Luciferase (Luc)-labeled SGC-7901 cells were preserved in our lab as previously described (1). The lentivirus pLV3-ECD shRNA that expresses ECD shRNA was purchased from GenePharma (Shanghai, China). The Luc-labeled SGC-7901 cells were transduced with lentivirus pLV3-ECD shRNA for 3 days, after which the SGC-7901-Luc-ECD shRNA-transduced cells were selected by 3 μg/ml puromycin

for two weeks. ECD silencing was validated by western blotting, and the effects of ECD silencing on migration and invasion were confirmed by transwell assay.

Immunofluorescence staining

SGC-7901 cells were co-transfected with Flag-hnRNP F (Flag-F) and HA-ZFP91 or Flag-ECD and HA-ZFP91 for 24 h, after which they were plated on glass cover slips. The cells were then fixed with 4% paraformaldehyde, permeabilized with 0.1% Triton X-100, and incubated with anti-ECD, hnRNP F, Flag or HA antibodies before being incubated with the appropriate Alexa Fluor 488 or Cy3-conjugated secondary IgG antibodies. The cell nuclei were stained with DAPI.

The siRNA sequences used in this study

gene	No.		sequences
ECD	1	Sense	5’-CCACAAGCAUUGAAUAUAATT-3’
		Anti-sense	5’-UUAUAUUCAAUGCUUGUGGTT-3’
	2	Sense	5’-GAGAUCUUAUGCUCCAAAUTT-3’
		Anti-sense	5’-AUUUGGAGCAUAAGAUCUCTT-3’
HnRNP F	1	Sense	5’-CCGCAGGUGUCCAUUUCAUTT-3’
		Anti-sense	5’-AUGAAAUGGACACCUGCGGTT-3’
	2	Sense	5’-GGUACAUUGAGGUGUUCAATT-3’
		Anti-sense	5’-UUGAACACCUCAAUGUACCTT-3’
ZFP91	1	Sense	5’-CAUUGCUGCAUCUAGACCUTT-3’
		Anti-sense	5’-AGGUCUAGAUGCAGCAAUGTT-3’
	2	Sense	5’-GCAGCUCAUUUGCAAGUCATT-3’
		Anti-sense	5’-UGACUUGCAAAUGAGCUGCTT-3’
Negative Control	1	Sense	5’-GCACAAGCUGGAGUACAACUACATT-3’
		Anti-sense	5’-UGUAGUUGUACUCCAGCUUGUGCTT-3’

The primers used in this study

Primers name		Sequence (5’-3’)
Flag-ECD	Forward	CACCCCGGGCGAAGAAACCATGAAGCTTGC
	Reverse	CGCTCTAGATAAACGGGCCCTCTAGATGTGC
Flag-hnRNP F	Forward	AACTGCAGACCTGACACCCACCCTGAC
	Reverse	GCTCTAGATTGGCTGCCTGTGAAAATGA
HA-hnRNP F	Forward	CGCGGATCC ACCTGACACCCACCCTGAC
	Reverse	GCTCTAGATTAAGCGTAATCTGGAACATCGTATGG GTA GTCATAGCCACCCATGCTGT
Flag-ZFP91	Forward	CGCGGATCCCCGGGGGAGACGGAAGAGCC
	Reverse	CCGCTCGAGCGGCGAAGGTCCGGCAGAGTCTG
HA-ZFP91	Forward	CGGGGTACCCCGGGGGAGACGGAAGAGCC
	Reverse	CGGAATTCTTAAGCGTAATCTGGAACATCGTATG GGTAAGGTCCGGCAGAGTCTG
hnRNP F (RT-PCR)	Forward	TGCCCTGGTCCTGCTCTGTT
	Reverse	CCCAATCCATCTCGGTTCTG
GAPDH (RT-PCR)	Forward	GAAGGTGAAGGTCGGAGTC
	Reverse	AAGATGGTGATGGGATTTC
Flag-ECD △C262	Forward	CACCCCGGGCGAAGAAACCATGAAGCTTGC
	Reverse	CGCTCTAGATTAAGGGCTCATAGCAAGAGAAC
Flag-ECD △C48	Forward	CACCCCGGGCGAAGAAACCATGAAGCTTGC
	Reverse	CGCTCTAGATTAGTCTACATCTACTGGTGCCA
Flag-ECD △N596	Forward	CACCCCGGGCGGTGAAGAAATCTTAACCTTATTACA
	Reverse	CGCTCTAGATTAGTCTACATCTACTGGTGCCA
Flag-ECD △N382	Forward	CGCTCTAGATTAGTCTACATCTACTGGTGCCA
	Reverse	CGCTCTAGATAAACGGGCCCTCTAGATGTGC
Flag-hnRNP F K87R	Forward	CCGGTACATTGAGGTGTTCAGGTCCCACAG
	Reverse	CTGTGGGACCTGAACACCTCAATGTACCGG
Flag-hnRNP F	Forward	CTCGCAGGAGTTAGCTGAGAGGGCTCTAGG

K167R	Reverse	CCTAGAGCCCTCTCAGCTAACTCCTGCGAG
Flag-hnRNP F K171R	Forward	AGCTGAGAAGGCTCTAGGGAGACACAAGGA
	Reverse	TCCTTGTGTCTCCCTAGAGCCTTCTCAGCT
Flag-hnRNP F K185R	Forward	GGTACATTGAGGTGTTTAGGAGCAGCCAGG
	Reverse	CCTGGCTGCTCCTAAACACCTCAATGTACC

A list of antibodies used for Western blot, IHC staining

Name of anti-body	Cat.No	Company	Mol weight	Dilution (WB/IHC)
ECD	10192-1-AP	Proteintech	73kD	1:1000(WB)
ECD	HPA006465	Sigma-Aldrich	73kD	1:500(IHC)
hnRNP F	14974-1-AP	Proteintech	46kD	1:1000(WB)
hnRNP F	ab50982	Abcam	46kD	1:5000(IHC)
Flag	M185-3L	MBL	Tag antibody	1:2000
HA	561	MBL	Tag antibody	1:2000
ZFP91	PA5-41199	ThermoFisher	63kD	1:2000
β-actin	60008-1-Ig	Proteintech	42kD	1:4000

References

1. Xu SH, *et al.* ACK1 promotes gastric cancer epithelial-mesenchymal transition and metastasis through AKT-POU2F1-ECD signalling. J Pathol **2015**;236:175-85
